# Targeting Programmed Cell Death in Acquired Sensorineural Hearing Loss: Ferroptosis, Necroptosis, and Pyroptosis

**DOI:** 10.1007/s12264-025-01370-y

**Published:** 2025-04-22

**Authors:** Shasha Zhang, Hairong Xiao, Yanqin Lin, Xujun Tang, Wei Tong, Buwei Shao, He Li, Lei Xu, Xiaoqiong Ding, Renjie Chai

**Affiliations:** 1 State Key Laboratory of Digital Medical Engineering, Department of Otolaryngology-Head and Neck Surgery, Zhongda Hospital, School of Life Sciences and Technology, Advanced Institute for Life and Health, Jiangsu Province High-Tech Key Laboratory for Bio-Medical Research, Southeast University, Nanjing, 210096 China; 2 Southeast University Shenzhen Research Institute, Shenzhen, 518063 China; 3 Co-Innovation Center of Neuroregeneration, Nantong University, Nantong, 226001 China; 4 Department of Neurology, Aerospace Center Hospital, School of Life Science, Beijing Institute of Technology, Beijing, 100081 China; 5 Department of Otolaryngology Head and Neck Surgery, Sichuan Provincial People’s Hospital, School of Medicine, University of Electronic Science and Technology of China, Chengdu, 610072 China; 6 Institute for Stem Cells and Regeneration, Chinese Academy of Science, Beijing, 100081 China; 7 Department of Otolaryngology-Head and Neck Surgery, Shandong Provincial ENT Hospital, Shandong University, Jinan, 250022 China; 8 Department of Otolaryngology, The First Affiliated Hospital of Wenzhou Medical University, Wenzhou, 325000 China; 9 School of Medicine, Faculty of Medical & Health Sciences, Tel Aviv University, 6997801 Tel Aviv, Israel

**Keywords:** Sensorineural hearing loss, Programmed cell death, Ferroptosis, Necroptosis, Pyroptosis

## Abstract

Sensorineural hearing loss (SNHL), the most commonly-occurring form of hearing loss, is caused mainly by injury to or the loss of hair cells and spiral ganglion neurons in the cochlea. Numerous environmental and physiological factors have been shown to cause acquired SNHL, such as ototoxic drugs, noise exposure, aging, infections, and diseases. Several programmed cell death (PCD) pathways have been reported to be involved in SNHL, especially some novel PCD pathways that have only recently been reported, such as ferroptosis, necroptosis, and pyroptosis. Here we summarize these PCD pathways and their roles and mechanisms in SNHL, aiming to provide new insights and potential therapeutic strategies for SNHL by targeting these PCD pathways.

## Introduction

According to the WHO (https://www.who.int/news-room/fact-sheets/detail/deafness-and-hearing-loss), >5% of the world’s population (~430 million people) suffer from disabling hearing loss (HL), including 34 million children, and it is estimated that >700 million people will have disabling HL by 2050. Sensorineural HL (SNHL) is the most commonly-occurring form of HL, and it is primarily caused by the degeneration, damage, or loss of sensory hair cells (HCs) and spiral ganglion neurons (SGNs) [1]. In adult mammals, HCs and SGNs lack the capacity for spontaneous regeneration and thus cannot replenish lost HCs and SGNs [2, 3], which leads to permanent SNHL. Therefore, it is necessary to study the mechanisms of SNHL and identify targets for HC and SGN protection in order to develop therapeutic strategies. Acquired SNHL can be caused by many environmental and physiological factors, such as ototoxic drugs, noise exposure, aging, bacterial and viral infection, and some diseases [1]; among these factors, ototoxic drugs, noise exposure, and aging have been extensively studied.

Ototoxic drugs, including aminoglycoside antibiotics and chemotherapeutic drugs, are among the most common factors that induce HL in the clinic when used for treating other diseases. Aminoglycosides, such as neomycin, gentamicin, streptomycin, and kanamycin, are used in the clinic as antibiotics against Gram-negative bacteria, some specific Gram-positive bacteria, and *Pseudomonas*, but these drugs can cause nephrotoxicity, ototoxicity, and to a lesser extent neuromuscular toxicity [4]. Aminoglycosides can enter the endolymph of the inner ear through the stria vascularis (SV), and this occurs mainly through ion channels, endocytosis, and transporter proteins [5]. Mechanoelectrical transduction (MET) channels, which are nonspecific cation channels in the tips of HC stereocilia, are the main pathway through which aminoglycosides enter HCs, whereupon they cause HC toxicity and aminoglycoside-induced HL [6, 7].

Cisplatin and carboplatin are platinum-based chemotherapeutic drugs that have broad-spectrum anti-tumor effects, especially against childhood cancers, but they also have side-effects such as ototoxicity, nephrotoxicity, and neurotoxicity [8, 9]. Cisplatin can cause HC death, SGN degeneration, and SV injury, which leads to cisplatin-induced HL [10]. Cisplatin enters cochlear HCs not only through MET channels similar to aminoglycosides, but also through copper transporter 1 (CTR1), CTR2, copper transporting p-type ATPase 1 (ATP7A), ATP7B, and organic cation transporter 1 (OCT1), and OCT2 [11–13].

Noise exposure – depending on its intensity and duration – can cause a reversible temporary threshold shift (TTS) that resolves over days or weeks or can cause a permanent threshold shift (PTS) that is irreversible [14]. Moderate-intensity noise exposures can damage the hair bundles of HCs and the ribbon synapses connecting HCs and SGNs, which may lead to a TTS resolving within days or weeks as these injuries heal. However, when intense and/or long-term noise exposure leads to massive loss of HCs and SGNs and greater damage to the SV, there can be a PTS and the development of irreversible noise-induced HL (NIHL) [15, 16].

Age-related HL (ARHL, also called presbycusis) is progressive, irreversible, bilateral, and symmetrical and develops gradually with age with multiple complex factors inducing its onset and severity [17]. ARHL is often caused by a combination of multiple factors and has been reported to have a certain relationship with tinnitus, Alzheimer's disease, cognitive decline, and late-life depression [18, 19]. Sensory ARHL is caused only by the degeneration of HCs, striatal ARHL is caused by atrophy of the SV, and neurologic ARHL is caused by the degeneration of the auditory nerve; SGNs belong to the SNHL category [20].

Many other factors can cause SNHL, and in most cases, accumulation of reactive oxygen species (ROS), increased inflammation, and mitochondrial damage can be detected in HCs, SGNs, and/or the SV, which leads to SNHL and cell death [5, 8, 14, 20]. Apoptosis, the first identified form of programmed cell death (PCD) [21], is the most extensively studied PCD pathway that contributes to the death of HCs and SGNs in SNHL [22, 23]. The factors noted above – ototoxic drugs, aging, and noise exposure – can induce ROS accumulation and production of cell death ligands, which directly induce intrinsic and extrinsic apoptosis *via* caspase-dependent and independent pathways, and therefore lead to SNHL. Autophagic cell death was the next PCD pathway identified after apoptosis [24], and it has also been reported to be involved in SNHL. Autophagy is a cellular process serving as a recycling system for cell self-degradation and re-utilization. Autophagy promotes cell survival and plays an antioxidative role in SNHL, but excessive autophagy can result in autophagic cell death, leading to subsequent SNHL [25–27].

**Fig. 1:**
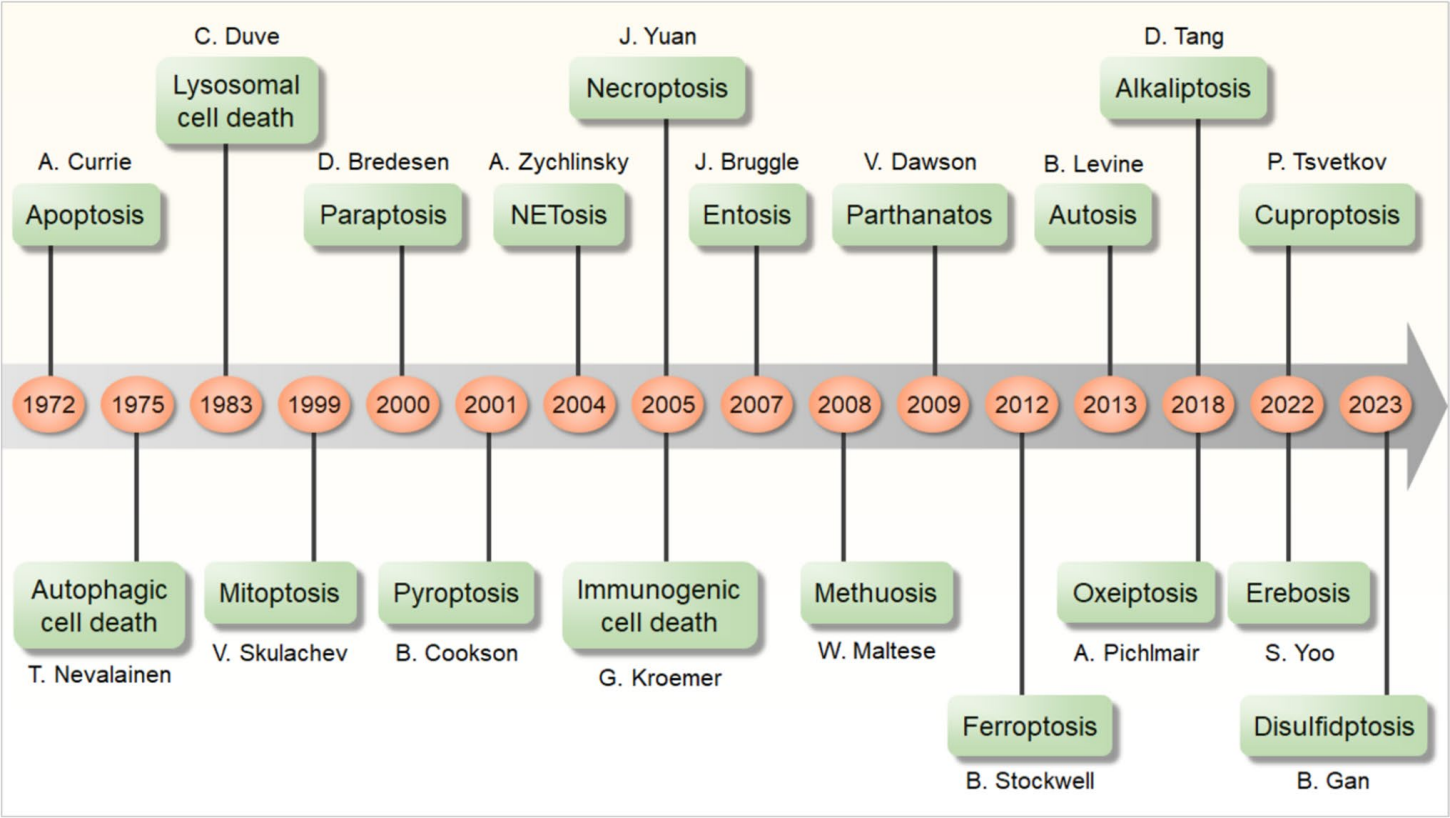
Discovery timeline of programmed cell death. From 1972 to 2023, 19 kinds of programmed cell death were discovered. The years of discovery are labeled in orange circles, and the names of programmed cell death are labeled in green boxes.

After apoptosis and autophagic cell death were discovered, an increasing number of novel forms of PCD pathways have been found and defined, including disulfidptosis [28], erebosis [29], cuproptosis [30], oxeiptosis [31], alkaliptosis [32], autosis [33], ferroptosis [34], parthanatos [35], methuosis [36], entosis [37], immunogenic cell death [38], necroptosis [39], NETosis [40], pyroptosis [41], paraptosis [42], mitoptosis [43], and lysosomal cell death [44], which have been found to play roles in tumors, degenerative diseases, and tissue injuries (Fig. 1). Among them, ferroptosis, pyroptosis, and necroptosis are all recently-defined PCD pathways that have been reported to play roles in SNHL. Here we summarize the mechanisms of these three PCD pathways and recent studies on PCD pathways in SNHL.

## Ferroptosis and SNHL

### Molecular Mechanism of Ferroptosis

**Fig. 2:**
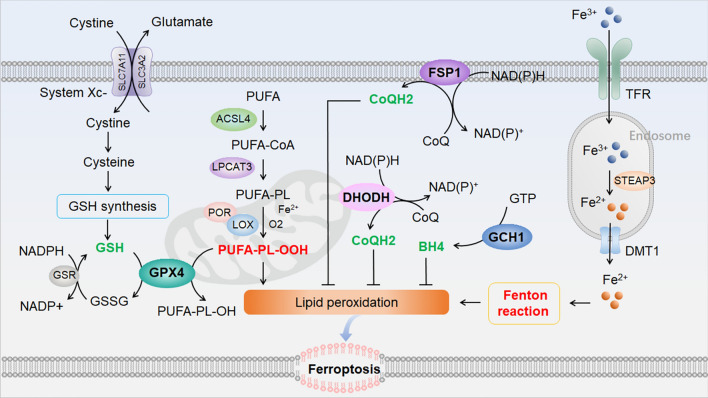
The molecular mechanism of ferroptosis. Ferroptosis is caused by excessive lipid peroxidation. PUFA-PL-OOHs are ferroptosis executioners that can be deoxygenated from PUFA-PLs or induced by the Fenton reaction. There are four main cellular antioxidant systems to suppress ferroptosis by reducing lipid peroxidation: the GSH-GPX4 system, the CoQH2-FSP1 system, the CoQH2-DHODH system, and the BH4-GCH1 system. Abbreviations: ACSL4, acyl-CoA synthetase long-chain family member 4; BH4, tetrahydrobiopterin 4; CoQ, oxidized coenzyme Q (ubiquinone); CoQH2, reduced coenzyme Q (ubiquinol); DHODH, dihydroorotate dehydrogenase; DMT1, divalent metal transporter 1; FSP1, ferroptosis suppressor protein 1; GCH1, GTP cyclohydrolase 1; GTP, guanosine triphosphate; GPX4, glutathione peroxidase 4; GSH, glutathione; GSSG, oxidized glutathione; LOX, lipoxygenase; LPCAT3, lysophosphatidylcholine acyltransferase 3; NAD^+^, the oxidized form of nicotinamide adenine dinucleotide; NADH, the reduced form of nicotinamide adenine dinucleotide; NADP^+^, the oxidized form of nicotinamide adenine dinucleotide phosphate; NADPH, the reduced form of nicotinamide adenine dinucleotide phosphate; POR, cytochrome P450 oxidoreductase; PUFA, polyunsaturated fatty acid; PUFA-CoA, polyunsaturated fatty acyl CoA; PUFA-PL, polyunsaturated fatty acyl phospholipid; PUFA-PL-OOH, PUFA-PL hydroperoxides; STEAP3, the six-transmembrane epithelial antigen of prostate 3.

Ferroptosis (Fig. 2) is an iron-dependent PCD pathway caused by excessive lipid peroxidation [34]. Ferroptosis has been well studied and is involved in several diseases including ischemic injury, cancer, degenerative diseases, inflammation, and infection [45–47]. Cells undergoing ferroptosis show increased membrane density, smaller mitochondria, and rupture of the outer mitochondrial membrane, but without the characteristic morphological features of apoptosis, necrosis, and autophagy [34, 48]. The hallmark of ferroptosis is unrestrained peroxidation of polyunsaturated fatty acyl phospholipids (PUFA-PLs), which occurs through both enzymatic as well as non-enzymatic mechanisms. In the enzymatic pathway, PUFA-PLs are deoxygenated into ferroptosis executioner PUFA-PL hydroperoxides (PUFA-PL-OOHs) by lipoxygenases (LOXs) and cytochrome P450 oxidoreductase (POR) [49–53]. PUFA-PLs are synthesized from PUFA through ligation of coenzyme A (CoA) by acyl-CoA synthetase long-chain family member 4 (ACSL4) and then re-esterification by lysophosphatidylcholine acyltransferase 3 [54–56]. The non-enzymatic pathway is associated with the iron-dependent Fenton reaction. Transferrin (TF)-bound Fe^3+^ is transferred by the TF receptor into cells, and then reduced to Fe^2+^ by the Six-Transmembrane Epithelial Antigen of the Prostate 3 in the endosome [57]. Excess Fe^2+^ is transported by divalent metal transporter 1 into the cytoplasm, and this results in the Fenton reaction, which induces lipid peroxidation and yields PUFA-PL-OOHs [58, 59]. The term ferroptosis comes from its dependence on iron, which is not only because the Fenton reaction involves iron, but also because the enzyme activity of LOXs and POR requires iron.

Several cellular antioxidant systems have been found to suppress ferroptosis, including the reduced glutathione (GSH) - glutathione peroxidase 4 (GPX4) system [60, 61], the reduced coenzyme Q (CoQH2, also called ubiquinol) - ferroptosis suppressor protein 1 (FSP1) system [62, 63], the CoQH2 - dihydroorotate dehydrogenase system [64], and the tetrahydrobiopterin - GTP cyclohydroxylase 1 system [65, 66]. Among them, the GPX4-GSH system was the first studied and is the most well-known antioxidant pathway regulating canonical ferroptosis. GPX4, a selenoprotein, inhibits ferroptosis by catalyzing the reduction of PL-OOHs to yield PL-alcohols with GSH as a cofactor [67–69]. Cysteine is the rate-limiting precursor of GSH, and cells take up its oxidized form cystine *via* the Xc^-^ antiporter system [70–73]. Deficiency or inactivation of GPX4 leads to deprivation of cysteine, which results in a decrease in the antioxidant GSH and subsequent PL-OOH accumulation, which induces ferroptosis [45]. FSP1, also known as AIFM2, is a GSH-independent ferroptosis suppressor that acts in parallel with GPX4 [62, 63]. FSP1 acts as an oxidoreductase that reduces CoQ (also called ubiquinone) to CoQH2, which traps lipid peroxyl radicals and thus suppresses lipid peroxidation and ferroptosis.

### Targeting Ferroptosis in SNHL

Several recent studies have reported the roles of ferroptosis in HL and HC damage *in vitro* and *in vivo*, and ferroptosis is involved in the ototoxicity caused by cisplatin [74–80], neomycin [81], noise exposure [82], aging [83–85], hydroperoxides [82, 86], and free fatty acids (FFAs) [87]. Among these, the roles of ferroptosis in cisplatin-induced ototoxicity are the most studied. The typical features of ferroptosis, such as lipid peroxidation, iron accumulation, and increased ROS generation, have been reported in HEI-OC1 (House Ear Institute-Organ of Corti 1) cells, cochlear explants, and cochleae of mice after treatment with cisplatin [74, 76–79]. The canonical GPX4 antioxidant system is reported to be involved in this process, and GPX4, ACSL4, and SLC7A11 all decrease in response to cisplatin treatment [77–79]. In a recent study, several key ferroptosis regulator genes, such as Gpx4 and Fsp1, were altered in cisplatin-damaged cochlear explants based on RNA sequencing, implying the induction of ferroptosis. *In vivo* studies have shown that hearing impairment, severe outer HC (OHC) loss, and progressive damage of synapses in inner HCs (IHCs) occur in Atoh1-Gpx4-/- mice (Gpx4 HC conditional knockout mice), but not in Fsp1 KO mice, which indicates that Gpx4, but not Fsp1, plays an important role in the functional maintenance of HCs [75]. In cisplatin-induced SGN damage, ferroptosis and lipid peroxidation are induced *via* FOXO1-NCOA4 axis-mediated ferritinophagy, and inhibiting ferroptosis can protect SGNs from cisplatin-induced damage and HL [88].

Several studies have also found some drugs and regulatory targets that prevent HC damage and HL by targeting ferroptosis (Table 1). Ferrostatin-1 (Fer-1) is a well-known ferroptosis inhibitor that acts as a radical-trapping antioxidant, and it can effectively alleviate cisplatin-induced ototoxicity [74–77], noise-induced HL [82], and FFA-induced ferroptosis [87]. Fer-1 can protect from cisplatin damage to HCs and hearing function by reducing intracellular and mitochondrial Fe^2+^, reducing the mitochondrial ROS level, reducing the GSH/GSSG ratio, inducing GPX4 expression, and protecting against mitochondrial damage [76, 77]. Fer-1 protects from noise-induced HL and loss of OHCs, ribbon synapses, and auditory nerve fibers by reducing lipid peroxidation, decreasing ROS levels, and inhibiting TFR1 [82]. Liproxstatin-1, also a ferroptosis inhibitor acting as a radical-trapping antioxidant [89], has also been reported to alleviate the neomycin-induced increase in ROS generation [81]. 4-Octyl itaconate (4-OI), a cell-permeable and anti-inflammatory/anti-oxidant metabolite derived from itaconic acid, inhibits cisplatin-induced ferroptosis and cell loss in cochlear explants and HEI-OC1 cells [78]. 4-OI can protect against cisplatin damage to HCs by inducing SLC7A11 and GPX4 expression levels and activating the NRF2/HO-1 signaling pathway. Deferoxamine (DFO), an iron chelator widely used to reduce iron accumulation and deposition, has been reported to inhibit ferroptosis in several disease models [90, 91]. DFO attenuates tert-butyl hydroperoxide-induced damage *in vitro* cochlear explants and HEI-OC1 cells by reducing iron accumulation and lipid peroxidation, consequently alleviating ferroptosis and apoptosis [86]. CMS121 protects against inflammation and excess lipid peroxidation by acting as a fatty-acid synthase inhibitor [92]. SAMP8 mice (a mouse model of ARHL) treated with CMS121 show a significant reduction in threshold shifts of the auditory brainstem response (ABR) and increased IHC ribbon synapse protection [85]. Luteolin, an FDA-approved drug that can be applied in clinical practice, was recently shown to specifically inhibit ferroptosis and thus alleviate cisplatin-induced ototoxicity *via* decreasing transferrin expression and intracellular Fe^2+^ concentration [75]. Recently, a conductive slow-release hydrogel-based drug cocktail delivery system has been developed to protect HCs from oxidative stress and various forms of cell death, including ferroptosis [93]. Among these drugs, Fer-1 is the most extensively studied and widely used in ferroptosis inhibition, and Luteolin is the only FDA-approved drug that has been reported to have no side-effects [75]. The side-effects of other drugs remain to be elucidated.

**Table 1:** Drugs or compounds that alleviate SNHL by targeting PCD pathways.

Targeted PCD pathways	Drugs or compounds	Molecular formula / CAS No.	Mechanisms	Damage factors causing SNHL	Object of study	Effect/side-effect	Reference
Ferroptosis	Ferrostatin-1 (Fer-1)	C_15_H_22_N_2_O_23_ 47174-05-4	Ferroptosis inhibitor / radical-trapping antioxidant	Cisplatin	HEI-OC1 cells Cochlear explants Zebrafish C57BL/6 mice Atoh1-Gpx4−/− mice	HEI-OC1 cell viability increase HC protection ABR/DPOAE threshold decrease Intracellular Fe^2+^ and mitochondrial Fe^2+^ decrease ROS level decrease GSH/GSSG ratio increase Protects against mitochondrial damage GPX4 increase	[74–77]
				Noise	CBA/J mice	ABR threshold decrease HC protection Ribbon synapse protection Auditory nerve fiber protection ROS level decrease TFR1 inhibition	[82]
	Liproxstatin-1 (Lip-1)	C_19_H_21_ClN_4_ 950455-15-9	Ferroptosis inhibitor / radical-trapping antioxidant	Neomycin	HEI-OC1 cells Cochlear explants	HEI-OC1 cell viability increase HC protection	[81]
	4-Octyl itaconate (4-OI)	C_13_H_22_O_4_ 3133-16-2	Anti-inflammatory / anti-oxidant metabolite	Cisplatin	HEI-OC1 cells Cochlear explants C57BL/6J mice	HEI-OC1 cell viability increase HC protection SLC7A11 and GPX4 increase Activates NRF2/HO-1 signaling pathway ABR threshold decrease	[78]
	Deferoxamine (DFO)	C_25_H_48_N_6_O_8_ 70-51-9	Iron chelator	Tert-butyl hydroperoxide	HEI-OC1 cells Cochlear explants	HC protection ROS level decrease Lipid peroxidation decrease TFR1 inhibition HEI-OC1 cell viability increase GSH/GSSG ratio increase Intracellular Fe^2+^ and mitochondrial Fe^2+^ decrease Activates Nrf2 signaling pathway	[86]
	CMS121	C_20_H_19_NO_3_ 1353224-53-9	Fatty acid synthase inhibitor	Aging	SAMP8 mice	ABR threshold decrease Ribbon synapse protection	[85]
	Luteolin	C_15_H_10_O_6_ 491-70-3 (FDA-approved)	Reduces transferrin expression and intracellular Fe^2+^ concentration	Cisplatin	HEI-OC1 cells C57BL/6 mice Pou4f3-Gpx4^Cre/ER^ mice	HC protection ABR threshold decrease Transferrin decrease Intracellular Fe^2+^ decrease / no side-effect	[75]
Necroptosis	Necrostatin-1 (Nec-1)	C_13_H_13_N_3_OS 4311-88-0	RIPK1 inhibitor	Ouabain	SGNs derived from fetal rats *in vitro* Sprague-Dawley rats	SGN protection pRIPK3 and pMLKL decrease ABR/DPOAE threshold decrease	[122, 123]
				Cisplatin	HEI-OC1 cells, C57BL/6 mice	ABR threshold decrease HC protection	[120, 121]
				Tunicamycin-induced ER stress	Cochlear explants HEI-OC1 cells	HEI-OC1 cell viability increase HC protection ROS level decrease Reduces IL-1β	[117]
				Virus infection	Cochlear explants	HC protection	[125]
				Kanamycin	C57BL/6 mice	ABR threshold decrease	[120]
	GW806742X (GW80)	C_25_H_22_F_3_N_7_O_4_S 579515-63-2	MLKL inhibitor	Tunicamycin-induced ER stress	C57BL/6J mice Cochlear explants HEI-OC1 cells	HEI-OC1 cell viability increase HC protection ROS level decrease Reduces IL-1β	[117]
	Ponatinib	C_29_H_27_F_3_N_6_O 943319-70-8	RIPK1 and RIPK3 inhibitor	Virus infection	Cochlear explants	HC protection	[125]
	Resveratrol	C_14_H_12_O_3_ 501-36-0	A natural polyphenol and phytoalexin	Aging	15-month-old C57BL/6 mice	ABR threshold decrease HC protection RIPK1, RIPK3, and pMLKL decrease	[118]
Pyroptosis	Oridonin (Ori)	C_20_H_28_O_6_ 28957-04-2	NLRP3 inhibitor	Noise	C57BL/6J mice	ABR threshold decrease Interrupts NLRP3-NEK7 binding IL-1β and IL-18 decrease	[155, 168]
				Kanamycin+ Furosemide	Kunming mice	ABR threshold decrease HC protection Inhibition of cleavage of Caspase-1 and GSDMD NLRP3, IL-1β, IL-6, and TNFα decrease	[160]
	MCC950	C_20_H_24_N_2_O_5_S 210826-40-7	NLRP3 inhibitor	Lipopolysaccharide	CAPS-associated NLRP3 mutant mice	ABR/DPOAE threshold decrease IL-1β decrease Alleviates mouse weight loss Inhibition of blood-labyrinth barrier disruption and macrophage infiltration	[165]
	Anakinra	C_759_H_1186_N_208_O_232_S_10_ 143090-92-0	IL-1β analog	Noise	C57BL/6J mice	ABR threshold decrease	[155]
	Piceatannol	C_14_H_12_O_4_ 10083-24-6	antioxidant / anti-inflammatory natural polyphenolic stilbene	Aging	48-week-old C57BL/6J mice HEI-OC1 cells	ABR threshold decrease HC and SGN protection Decrease of Caspase-11, GSDMD, Caspase-1, IL-1β, IL-18, NLRP3, NF-κB and ASC HEI-OC1 cell viability increase ROS level decrease	[156]
	BAY11-7082	C_10_H_9_NO_2_S 19542-67-7	Pyroptosis inhibitor	Aging	48-week-old C57BL/6J mice HEI-OC1 cells	ABR threshold decrease HC and SGN protection Decrease of Caspase-11, GSDMD, Caspase-1, IL-1β, IL-18. NLRP3, NF-κB and ASC ROS level decrease	[156]
	Melatonin	C_13_H_16_N_2_O_2_ 73-31-4	Neural antioxidant	Ischemia-reperfusion injury	HEI-OC1 cells Cochlear explants	HEI-OC1 cell viability increase ROS level decrease Decrease of GSDMD-N, Caspase-1, NLRP3, and ASC Increase of MT-1, MT-2, and Nrf2 ROS level decrease	[163]

## Necroptosis and SNHL

### Molecular Mechanism of Necroptosis

**Fig. 3:**
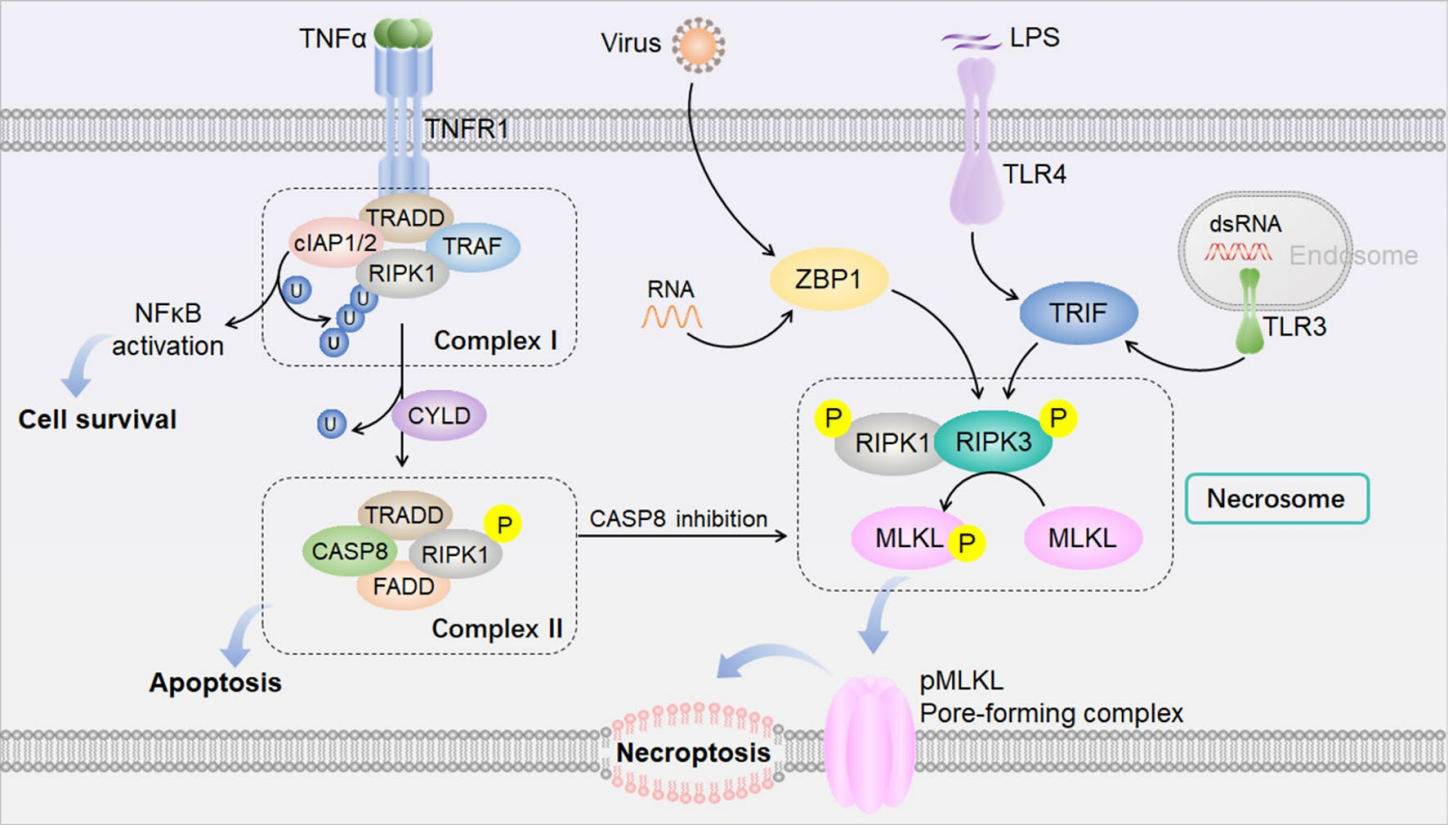
Molecular mechanism of necroptosis. Necroptosis is mediated by MLKL, which is phosphorylated by RIPK3 resulting in membrane pore formation. The canonical RIPK1-RIPK3-MLKL necrosome complex is induced mainly by death receptor pathways by forming Complexes I and II. When CASP8 in Complex II is blocked, RIPK1 is phosphorylated and interacts with RIPK3 to form a necrosome complex in which MLKL is phosphorylated by activated RIPK3. RIPK3 can also be activated by the RHIM domain-containing proteins TRIF and ZBP1 which are stimulated by viral infection, dsRNA, or LPS. Abbreviations: CASP8, cysteinyl aspartate-specific proteinase 8; cIAP, cellular inhibitors of apoptosis protein; CYLD, cylindromatosis; dsRNA, double-stranded RNA; FADD, Fas-associated death domain; LPS, lipopolysaccharide; MLKL, mixed lineage kinase domain-like protein; NFκB, nuclear factor kappa-B; RIPK1/3, receptor-interacting protein kinase 1/3; TNFα, tumor necrosis factor alpha; TNFR1, TNF receptor 1; TRADD, TNF receptor-associated death domain; TRAF, TNF receptor-associated factor; TRIF, TIR-domain-containing adapter-inducing interferon-β; TLR3/4, toll-like receptors 3/4; ZBP1, Z-DNA-binding protein 1.

Necroptosis (Fig. 3) is mediated by the pseudokinase mixed lineage kinase domain-like (MLKL), which is phosphorylated by receptor-interacting serine-threonine kinase 3 (RIPK3) resulting in membrane pore formation [94–96]. Necroptosis can be triggered by multiple intracellular and extracellular factors that stimulate tumor necrosis factor (TNF) receptor family proteins and Toll-like receptors (TLR) 3/4, and cells undergoing necroptosis show cell swelling, membrane rupture, and the release of intracellular material [97–101].

The canonical RIPK1-RIPK3-MLKL necrosome complex is induced mainly by death receptor pathways such as the TNF receptor (TNFR), the TNF-related apoptosis-inducing ligand (TRAIL), and Fas. When binding to TNFα, TNFR1 trimerizes, undergoes a conformational change, and recruits TNF receptor-associated factor 2, TNF receptor type 1-associated death domain (TRADD), RIPK1, and cellular inhibitors of apoptosis (cIAP) 1/2 to form Complex I [102, 103]. cIAPs within Complex I add ubiquitin chains to RIPK1, which is pivotal for the activation of nuclear factor kappa-B (NF-κB) to facilitate cell survival [104, 105]. Deubiquitylation of RIPK1 by deubiquitinase cylindromatosis leads to Complex II formation, which comprises TRADD, FAS-associated death domain protein, RIPK1, and caspase-8 [102]. When caspase-8 in Complex II is activated, RIPK1 is inactivated and apoptosis is induced [104, 106]. When caspase-8 is blocked, RIPK1 is phosphorylated and interacts with RIPK3 through its C-terminal RIPK homotypic interaction motif (RHIM) domain to form a necrosome complex in which MLKL is phosphorylated by activated RIPK3 [98, 107, 108]. Phosphorylated MLKL forms oligomers (pore-forming complexes), is translocated to the cell plasma membrane, and forms pores, which ultimately leads to necroptosis [109–113].

In addition, RIPK3 can also be activated by other stimuli and factors bypassing RIPK1. TIR-domain-containing adapter-inducing interferon-β (TRIF, also called TICAM1), and Z-DNA-binding protein (ZBP1, also called DAI), both of which are RHIM domain-containing proteins, can physically interact with RIPK3. ZBP1 can sense viral infections and activate RIPK3, thus inducing RIPK1-independent activation of necroptosis [101, 114, 115]. TRIF-dependent activation of RIPK3 can be induced by TLR3 and TLR4 activation which are stimulated by double-stranded RNA (dsRNA) and lipopolysaccharide (LPS), respectively, which ultimately result in necroptosis [100, 116].

### Targeting Necroptosis in SNHL

Necroptosis has been reported to be involved in ototoxicity, HL, and cochlear damage caused by aging [117–119], ototoxic drugs such as cisplatin and aminoglycosides [120, 121], the SGN-damaging drug ouabain [122, 123], noise exposure [124], viral infection [125], and ER stress [117, 126]. In almost all cases, canonical RIPK1-RIPK3-MLKL necroptosis is activated, which leads to HC or SGN damage and HL both *in vivo* and *ex vivo*. The expression and/or phosphorylation of RIPK1, RIPK3, and/or MLKL are increased in the aged mouse cochlea [119], cisplatin-treated organ of Corti (OC) and SGNs [121], ouabain-treated SGNs [122, 123], after noise exposure [124], and cochlear explants treated with the ER stress activator tunicamycin (TM) [117]. HEI-OC1 cells and cultured SGN cells have also been used as *in vitro* models to test the expression changes of RIPK1, RIPK3, and/or MLKL after damage [117, 121, 122]. RIPK3 inhibition protects against ouabain-induced HL and noise-induced necrotic OHC nuclei, while overexpression of RIPK3 aggravates ouabain-induced injury [122, 124]. In mice with viral infection-induced HL, virus-infected SCs and greater epithelial ridge cells produce TRAIL, which activates TRAIL death receptors 4 and 5, which are expressed in HCs and lead to HC necroptosis [125].

Several drugs are used to block necroptosis and thus protect HCs and SGNs from damage and mitigate HL (Table 1). Necrostatin-1 (Nec-1), a well-known RIPK1 inhibitor, has been extensively studied for targeting necroptosis to prevent HL. Nec-1 treatment can mitigate the HL caused by cisplatin and kanamycin [120], can protect HCs from damage by cisplatin [121], TM [117], and virus infections [125], and can protect SGNs from damage by ouabain and RIPK3 overexpression [122]. Nec-1 can protect against ouabain damage to SGNs and hearing function by reducing the phosphorylation of RIKP3 and MLKL [122]. Intraperitoneal injection of the MLKL inhibitor GW806742X (GW80) reduces the hearing threshold increase due to TM treatment to some extent, and *in vitro* and *ex vivo* treatment with GW80 inhibits the HC death induced by ER stress [117]. Ponatinib, which is reported to directly target and inhibit RIPK1 and RIPK3 [127], can protect HCs from damage by viral infections [125]. Resveratrol (RSV), a natural polyphenol and phytoalexin, can prevent ARHL by decreasing the expression levels of RIPK1, RIPK3, and MLKL in the cochlea, especially in the OC and SGNs [118]. Notably, only low-dose RSV (50 mg/kg) protects HCs and hearing function in ARHL. Moreover, some mixed treatments targeting necroptosis can also prevent HL. For example, treatment with Nec-1 and the pan-caspase inhibitor Z-VAD can decrease the ABR threshold shifts and increase SGN density after ouabain exposure [123], and in NIHL siRNA against RIPK3 combined with Z-VAD can reduce hearing thresholds more than Z-VAD treatment alone [124]. Among these drugs, Nec-1 is the most extensively studied and widely used in necroptosis inhibition. However, there is no study of the side-effect of all these necroptosis inhibition drugs, which should be studied in the future.

## Pyroptosis and SNHL

### Molecular Mechanism of Pyroptosis

**Fig. 4:**
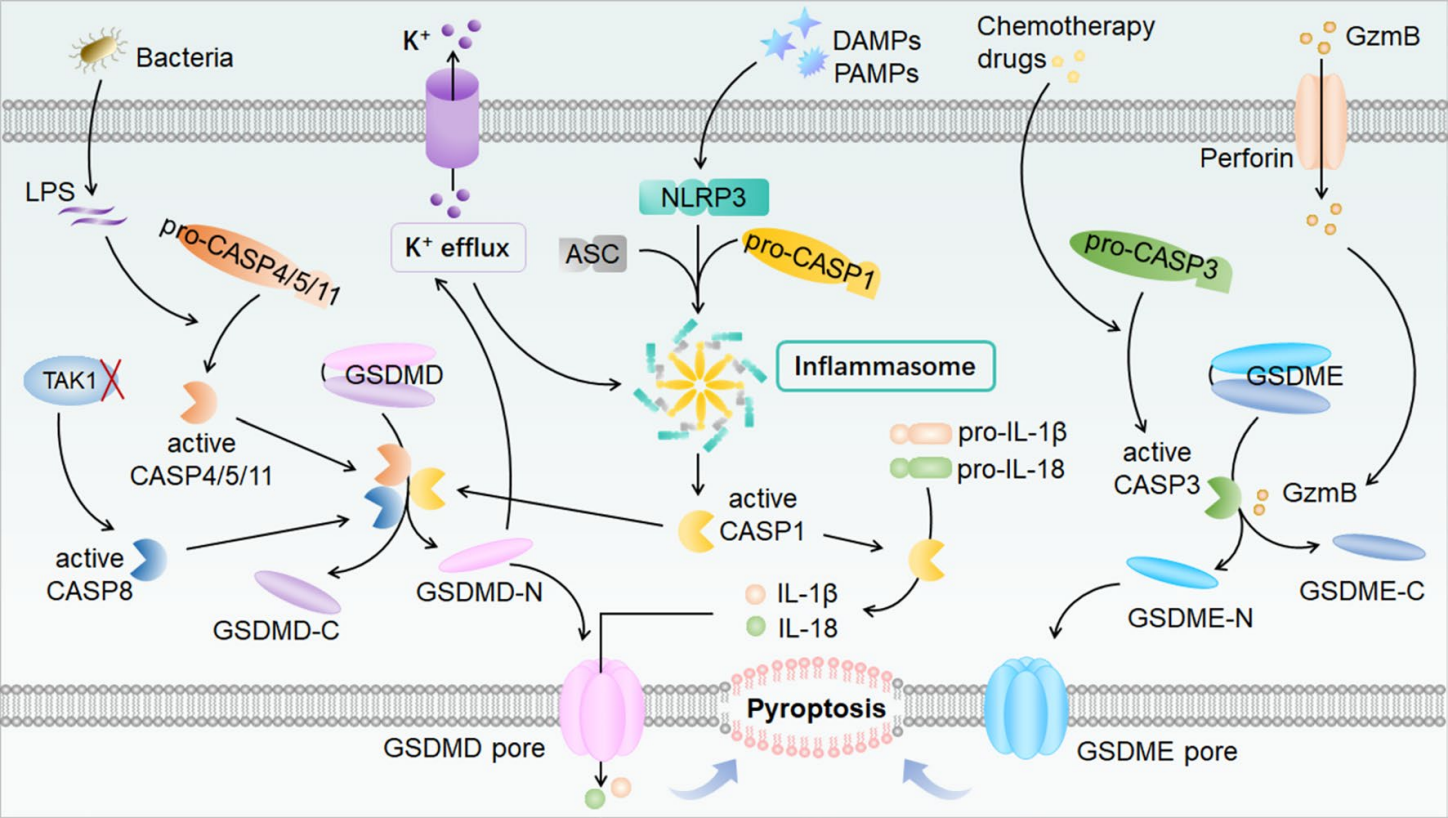
Molecular mechanism of pyroptosis. Pyroptosis is mediated by the cleaved N-terminus of GSDM proteins which are the executioners of pyroptosis and can form pores on the plasma membrane. Canonical pyroptosis is mediated by the inflammasome complex which cleaves and activates CASP1. CASP1 then cleaves and activates GSDMD, IL-1β, and IL-18, allowing GSDMD N-terminal membrane pore-formation and release of IL-1β and IL-18. Non-canonical pyroptosis is triggered by bacterial LPS which activates CASP-4/5/11 and then cleaves GSDMD without assembling the inflammasome. CASP-3 and CASP-8 can also cleave GSDME and GSDMD respectively to form pores on the plasma membrane and induce pyroptosis. Abbreviations: ASC, apoptosis speck-like protein; CASP1/3/4/5/8/11, cysteinyl aspartate specific proteinases 1/3/4/5/8/11; DAMPs, damage‐associated molecular patterns; GSDMD, gasdermin D; GSDME, gasdermin E; GzmB, granzyme B; IL-1β, interleukin-1β; IL-18, interleukin-18; LPS, lipopolysaccharide; NLRP3, NOD-like receptor family pyrin domain containing 3; PAMPs, pathogen-associated molecular patterns. TAK1, TGFβ-activated kinase 1.

Pyroptosis (Fig. 4) is mediated by the membrane pore-forming gastrin (GSDM) protein family [128]. Cells undergoing pyroptosis mainly show swelling, membrane perforation, and the release of inflammatory factors [129, 130]. There are canonical and non-canonical pyroptosis pathways that differ in terms of inflammasome formation, caspase activation, and executioner GSDM protein cleavage [131].

GSDM proteins, which have pore-forming activity, are the executioners of pyroptosis [128, 132]. The GSDM family members include GSDMA/B/C/D/E, and pejvakin (PJVK) [131, 133]. Among them, GSDMD and GSDME are the most well-studied. Except for PJVK, all GSDM proteins contain two conserved domains: the N-terminal pore-forming domain (PFD) and the C-terminal repressor domain (RD). When cleaved by some enzymes such as caspase-1, the C-terminal RD can no longer block the N-terminal PFD and thus the PFD oligomerizes to form pores in the plasma membrane, leading to the release of inflammatory molecules and subsequent pyroptosis [133].

Canonical pyroptosis is mediated by the inflammasome, which is an assembled complex of pattern recognition receptors (PRRs), apoptosis-associated speck-like protein (ASC), and pro-caspase-1 [134, 135]. PRRs, acting as inflammasome sensors, bind and recognize pathogen-associated molecular patterns and damage-associated molecular patterns. Some components of the innate immune system – including NOD-like receptors (NLRs), Toll-like receptors (TLRs), RIG-I-like receptors, AIM2-like receptors (ALRs), and C-type lectin receptors, are PRRs [136, 137]. Some NLRs and ALRs have been reported to assemble canonical inflammasomes and activate caspase-1, such as the NLR family pyrin domain containing (NLRP) 1/3, AIM2, and pyrin. Among them, the NLRP3 inflammasome is the most extensively studied. NLRP3 contains three important domains: a leucine-rich repeat domain (LRR), a pyrin domain (PYD), and a nucleotide-binding oligomerization domain (NACHT). ASC, acting as an inflammasome sensor that contains a PYD and NLR family caspase activation and recruitment domain, recruits NLRP3 and pro-caspase-1 to assemble the inflammasome [138]. Pro-caspase-1 assembled in inflammasomes is cleaved to become the active form and acts as an inflammasome effector, which is then able to cleave GSDMD into its C-terminal and N-terminal fragments. Meanwhile, mature interleukin (IL)-1β and IL-18 are activated by cleaved-caspase-1, and then released through plasma membrane pores formed by oligomerized GSDMD N-terminal PFD. All of the above ultimately result in pyroptosis.

There are also non-canonical pyroptosis and other pyroptosis pathways. Caspase-4/5/11 can be activated by bacterial LPS and then can directly cleave GSDMD without assembling an inflammasome [139, 140]. Cleaved GSDMD oligomerizes to form pores in cell membranes, and activated GSDMD leads to K^+^ efflux, which further promotes the formation of NLRP3 inflammasomes resulting in IL-1β and IL-18 maturation and secretion [128, 141–143]. ​Caspase-3 and Caspase-8 are pivotal caspases mainly involved in apoptosis. However, some reports have shown that they also play roles in pyroptosis. Active caspase-3 has the ability to cleave GSDME in chemotherapy-induced pyroptosis [144–147], and active caspase-8 under conditions of TGFβ-activated kinase 1 inhibition can cleave GSDMD to trigger pyroptosis [148, 149]. In addition, GSDME can also be cleaved directly by granzyme B (GzmB) to induce pyroptosis [150]. In addition, GSDMB can be cleaved by GzmA [151] and GSDMC can be cleaved by active caspase-8 induced by TNFα [152], and these both can trigger pyroptosis.

**Fig. 5:**
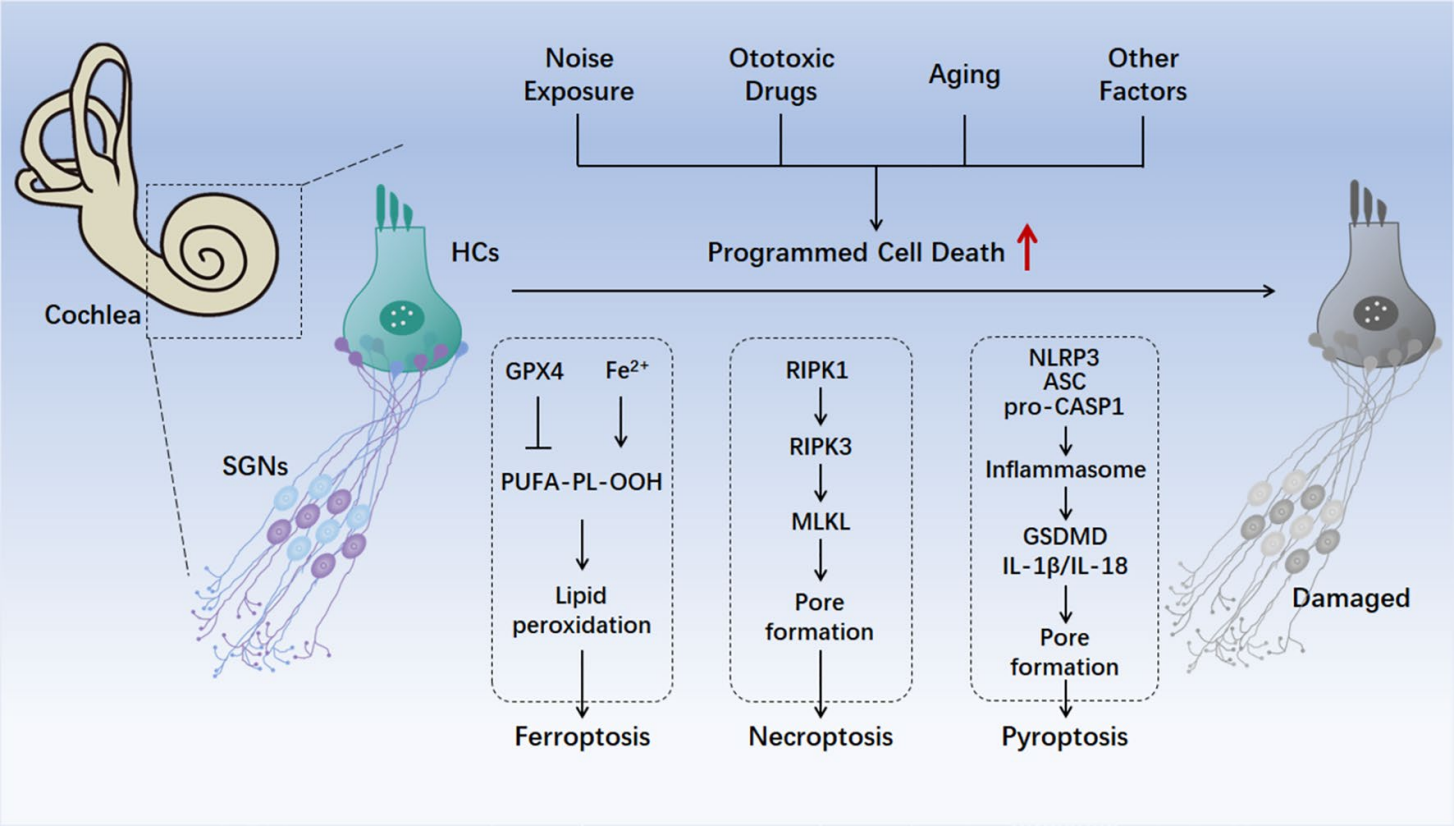
Main factors of three PCD pathways involved in SNHL. Many factors, such as ototoxic drugs, aging, and noise exposure can lead to PCD of cochlear HCs and SGNs, which is the main cause of SNHL. In addition to apoptosis and autophagic cell death, here are the other three PCD pathways – ferroptosis, necroptosis, and pyroptosis – that are involved in SNHL. Abbreviations: ASC, apoptosis speck-like protein; CASP1, cysteinyl aspartate specific proteinase 1; GPX4, glutathione peroxidase 4; GSDMD, gasdermin D; HCs, hair cells; PUFA-PL-OOH, PUFA-PL hydroperoxides; IL-1β, interleukin-1β; IL-18, interleukin-18; MLKL, mixed lineage kinase domain-like protein; NLRP3, NOD-like receptors family pyrin domain containing 3; RIPK1/3, receptor-interacting protein kinase 1/3; SGNs, spiral ganglion neurons.

### Targeting Pyroptosis in SNHL

Increasing numbers of reports show that pyroptosis is closely involved in SNHL. The most extensively studied inflammasome in SNHL is the NLRP3 inflammasome [153], which is involved in ototoxicity and the HL induced by numerous factors such as noise exposure [154, 155], aging [156, 157], cisplatin treatment [158, 159], kanamycin treatment [160], tumors [161], viral infections [162], oxygen-glucose deprivation/reperfusion [163], unconjugated bilirubin [164], and cryopyrin-associated periodic syndrome (CAPS)-associated NLRP3 mutations [165]. The expression of NLRP3, activated-caspase-1, IL-1β, and IL-18, are increased in the cochlea after noise exposure [154, 155], kanamycin injection [160], and cytomegalovirus infection [162], as well as in aging mice [157]. Pyroptosis and NLRP3 inflammasome activation have also been reported in cochlear marginal cells treated with cisplatin *in vitro* [159], human vestibular schwannoma [161], and oxygen-glucose deprivation/re-oxygenation (OGD/R)-treated HEI-OC1 cells [163]. Also, GSDMD is the main effector of pyroptosis in HL [157, 159, 160, 163, 164], while the Caspase-3/GSDME pathway, but not GSDMD, is activated in mouse cochlear HCs after cisplatin treatment [158]. Transgenic Nlrp3^D301NneoR/+^Gfi1^Cre/+^ mice have also been constructed to study pyroptosis in HL and showed that conditional mutant Nlrp3 activation in the cochlea causes mice to exhibit severe to profound HL [166].

Some therapeutic methods have also been studied based on NLRP3 inflammasome-induced pyroptosis (Table 1). Oridonin (Ori), a covalent NLRP3 inflammasome inhibitor [167], has been shown to ameliorate noise-induced HL and kanamycin + furosemide-induced HL [155, 160, 168]. Ori can protect against the HL caused by noise by interrupting the interaction between NLRP3 and NIMA-related kinase 7 (NEK7) and thus inhibiting downstream inflammasome activation [155]. Ori can protect against the damage to HCs and hearing function caused by kanamycin and furosemide treatment through inhibition of NLRP3/Caspase-1/DSDMD-induced inflammasome activation [160]. MCC950, an NLRP3-specific inhibitor directly blocking the NACHT domain [169, 170], significantly alleviates systemic LPS-induced HL in CAPS-associated NLRP3 mutant mice [165]. Anakinra, an IL-1β analog competitively binding and blocking the IL-1β receptor, can restore partial hearing in mice after noise exposure [155] Piceatannol, a natural polyphenolic stilbene with anti-tumor, antioxidant, and anti-inflammatory effects, and BAY11-7082, a previously identified NF-κB inhibitor that was recently shown to be a canonical inflammasome-induced pyroptosis inhibitor [171], can both protect against ARHL by attenuating cochlear pyroptosis and inflammation *via* regulating the caspase-11-GSDMD pathway [156]. Melatonin, a neural antioxidant [172], alleviates OGD/R-induced injury in HEI-OC1 cell cochlear explants by inhibiting pyroptosis *via* the MT-1/2/Nrf2/ROS/NLRP3 pathway [163]. Notably, the effects of melatonin are abolished or largely offset by the application of ML385 (an Nrf2 inhibitor) or luzindole (a non-selective melatonin receptor blocker). Among these drugs, Ori has been relatively extensively studied and is relatively widely used in pyroptosis inhibition. However, there are also no side-effect studies of any of these necroptosis inhibition drugs.

It is worth noting that three pivotal genes in pyroptosis – NLRP3, GSDMD, and DSDME – are also involved in hereditary non-syndrome deafness. NLRP3, also called deafness autosomal dominant 34 (DFNA34), was recently reported to cause progressive HL in two unrelated families by its missense mutation [173]. Mutations in GSDME (also referred to as DFNA5) have been found to cause autosomal-dominant non-syndrome deafness; the mutations result in the skipping of exon 8 and have a gain-of-function effect [174–178]. Hearing impairment caused by DFNA5 mutation is late-onset and progressive with impairment of high frequencies at first and later gradual extension to all frequencies [179, 180], but no hearing impairment was reported in Dfna5-knockout mice [181]. Pjvk, also called deafness autosomal recessive 59 (DFNB59), has been shown to cause autosomal-recessive non-syndromic deafness [182–184]. Pjvk plays a role in peroxisome proliferation, interacts with cytoskeletal proteins, and maintains stereocilia architecture in HCs [185–187], but whether Pjvk has the ability to form pores and is involved in pyroptosis remains unknown. Pjvk gene therapy mediated by AAV2/8 and N-acetyl cysteine treatment in Pjvk^–/–^ mice both provide hearing protection [185].

## Conclusion and Future Perspective

SNHL is a complex disease caused by many factors, and it lacks effective targeted treatment in the clinic. Inhibiting pathways that induce damage in the cochlea, especially damage of HCs and SGNs, prior to the onset of SNHL appears to be an effective and fundamental method for the prevention, alleviation, and treatment of SNHL. Therefore, it is necessary to study the mechanisms underlying HC and SGN cell death in SNHL. In addition to apoptosis and autophagy, an increasing number of studies have shown that the cell death of HCs and SGNs in SNHL can be caused by several recently-identified PCD pathways, including ferroptosis, necroptosis, and pyroptosis (Fig. 5). Here, we aim to summarize the mechanisms of three newly-discovered PCD pathways, reveal the latest research progress on their mechanisms and roles in SNHL, and summarize the effects of these PCD pathway inhibitors on SNHL and their target mechanisms, so as to provide hints and future perspective for subsequent research.

Among these PCD pathways, ferroptosis is mainly caused by excessive lipid peroxidation and cellular antioxidant systems are involved in suppressing ferroptosis, while cell death caused by necroptosis and pyroptosis both result from pore-formation by the MLKL complex and the GSDM complex. Caspase enzymes are involved both in apoptosis and pyroptosis, with inflammatory caspases (caspases 1/4/5/11) mainly involved in pyroptosis. Factors such as ototoxic drugs, aging, and noise exposure, are widely reported to lead to SNHL *via* inducing key factors in these PCD pathways. However, there is currently no research indicating which PCD pathway plays the most critical role in SNHL.

Many drugs have been reported to play protective roles by targeting these PCD pathways in SNHL, and here we have summarized some drugs targeting ferroptosis, necroptosis, or pyroptosis. However, SNHL cannot be fully inhibited and the protective effect of almost all of these drugs can only partially protect against cochlear damage. This may be because cell death in SNHL occurs through many PCD pathways, and blocking one pathway cannot fully prevent cell death, and because SNHL is not only caused by damage of HCs and SGNs, and damage to the SV, supporting cells, and many other cell types in the cochlea can also lead to SNHL.

Therefore, in the future, we believe that SNHL research should focus on the following areas. First, the roles and mechanisms of other newly-discovered PCD pathways should be studied to elucidate their roles in SNHL. There have been a few reports on this already. Cuproptosis, a very recently identified copper-dependent form of the PCD pathway, is reported to be involved in cisplatin-induced ototoxicity [188]. Parthanatos [189–191] and paraptosis [192] may also be involved in SNHL. Second, the detailed mechanisms and the PCD pathway network causing SNHL should be further studied to elucidate the specific roles and regulatory networks of various PCD pathways. This may be the major challenge in future studies, because of the complexity of inducing factors, the cell types involved, and the pathway regulation in SNHL. Third, based on mechanistic studies, different drug combinations of several PCD pathways should also be tested to identify the best treatment method for fully protecting against SNHL.
